# A multilevel investigation of inequalities in clinical and psychosocial outcomes for women after breast cancer

**DOI:** 10.1186/1471-2407-11-415

**Published:** 2011-09-28

**Authors:** Philippa H Youl, Peter D Baade, Joanne F Aitken, Suzanne K Chambers, Gavin Turrell, Christopher Pyke, Jeffrey Dunn

**Affiliations:** 1 Viertel Centre for Research in Cancer Control, Cancer Council Queensland, PO Box 201, Spring Hill, QLD, 4004, Australia; 2 Griffith Health Institute, Gold Coast Campus, Griffith University, QLD, 4222, Australia; 3 School of Public Health, Queensland University of Technology, Herston Road, Kelvin Grove, 4059, QLD, Australia; 4 University of Queensland Centre for Clinical Research, Brisbane, QLD, Australia; 5 Mater Breast Care Unit, Mater Hospital, Raymond Terrace, South Brisbane, QLD, 4001, Australia

## Abstract

**Background:**

In Australia, breast cancer is the most common cancer affecting Australian women. Inequalities in clinical and psychosocial outcomes have existed for some time, affecting particularly women from rural areas and from areas of disadvantage. We have a limited understanding of how individual and area-level factors are related to each other, and their associations with survival and other clinical and psychosocial outcomes.

**Methods/Design:**

This study will examine associations between breast cancer recurrence, survival and psychosocial outcomes (e.g. distress, unmet supportive care needs, quality of life). The study will use an innovative multilevel approach using area-level factors simultaneously with detailed individual-level factors to assess the relative importance of remoteness, socioeconomic and demographic factors, diagnostic and treatment pathways and processes, and supportive care utilization to clinical and psychosocial outcomes. The study will use telephone and self-administered questionnaires to collect individual-level data from approximately 3, 300 women ascertained from the Queensland Cancer Registry diagnosed with invasive breast cancer residing in 478 Statistical Local Areas Queensland in 2011 and 2012. Area-level data will be sourced from the Australian Bureau of Statistics census data. Geo-coding and spatial technology will be used to calculate road travel distances from patients' residence to diagnostic and treatment centres. Data analysis will include a combination of standard empirical procedures and multilevel modelling.

**Discussion:**

The study will address the critical question of: what are the individual- or area-level factors associated with inequalities in outcomes from breast cancer? The findings will provide health care providers and policy makers with targeted information to improve the management of women with breast cancer, and inform the development of strategies to improve psychosocial care for women with breast cancer.

## Background

Breast cancer is the most common cancer in women worldwide. It is estimated that 1.38 million women will be diagnosed with breast cancer in 2008 [1]. In Australia in 2007, 12, 567 women were diagnosed with breast cancer accounting for 27% of cancer diagnoses. Breast cancer is the second leading cause of cancer mortality (15% of all cancer deaths) overall, [2] and among women, it is the leading cause of premature mortality due to cancer [3]. Women diagnosed with breast cancer carry a significant psychological burden. Prevalence studies suggest that 33% of women with breast cancer will experience clinically significant psychological distress (e.g. anxiety and/or depression) that for many will persist over time, [4] and approximately one-third report moderate to high unmet supportive care needs after their diagnosis [5].

### Inequalities in survival, recurrence, treatment and psychosocial outcomes

There is a consistent rural gradient in breast cancer incidence, being higher in major cities, and lower in more rural areas [6-8]. In contrast survival tends to be lower for women living in more rural areas. In Queensland Australia, relative survival from breast cancer was 14% lower among women from inner regional areas, 24% lower in outer regional areas and 41% lower in remote areas compared to the major city area with similar patterns observed in other Australian states [9,10]. Patterns of treatment also vary geographically with women living in rural and remote areas up to 70% more likely to receive a mastectomy than breast conserving surgery, independent of disease stage [11]. Possible factors contributing to this differential include the need for women in rural areas to travel greater distances to radiotherapy services and lack of local access to tertiary hospitals [11,12]. Additionally, there is some evidence to suggest that rural women may perceive, or actually have, less control over treatment decisions, and this may be due to limited access to information about options for breast cancer treatment [13]. Emerging evidence suggests that rural cancer patients experience greater psychological morbidity than do their urban counterparts [14] and rural women report more unmet needs for help in dealing with fears about cancer recurrence [5].

### Possible reasons for observed inequalities in outcomes

#### Stage

Differences in stage are a possible reason for the observed differences in breast cancer survival. Cancer stage is the most strongly predictive of all clinical prognostic factors: 5-year relative survival rates vary from about 98% for women with tumours ≤ 10 mm to 73% for women with tumours 30 mm+ and 49% for those women with tumours of unknown size [10]. However a recent ecological study in Queensland, Australia found significant differences in 5-year survival by rurality that remained after adjusting for spread of disease, [10] suggesting these differences in survival are not explained by geographical variation in the proportions of women diagnosed with early versus late stage breast cancer.

#### Age

There is biological evidence to suggest that breast cancer among younger women is a distinct disease [15]. Tumours tend to be larger, less well differentiated, more likely to be lymph-node positive and more likely to metastasise compared to tumours found in older women. As a result, prognosis is significantly worse among younger breast cancer patients, and they are more likely to experience recurrence [15-17]. Compared to older women, women less than 50 years of age are more likely to experience high levels of psychological distress after breast cancer and greater ongoing psychosocial support needs [18]. Younger women also experience more problems with sexual well-being after breast cancer, [19] including concerns about premature menopause; fertility and sexual functioning; [20] greater decrements in social well-being; greater financial difficulties; [21] and they consistently report that existing supportive care services do not match their needs [22].

#### Risk factors

Apart from age and family history, risk factors for breast cancer include reproductive or hormonal factors, [23] as well as health-related factors such as obesity, smoking, alcohol intake and reduced levels of physical activity [24-26]. Those living in rural areas and areas of disadvantage are more likely to have poorer health-related behaviours [27]. Further, a high body mass index and current smoking have been found to be associated with reduced survival [28].

#### Access to screening and health services

Screening for breast cancer in asymptomatic women is estimated to reduce mortality by 20% to 35% [29,30]. In Australia, free biennial population-based screening is available for women in the target age group of 50 to 69 years, although women aged 40-49 years and over 70 years are also able to take part. Approximately 55% of Australian women in the target age group participated in the breast screen program in 2007-2008 [31]. While higher participation rates have been observed in regional and remote areas compared to major cities, low participation rates have been observed in the most socially disadvantaged areas and among Indigenous women [32].

Location of and access to, health services have been recognised as important contributors to morbidity and mortality in Australia and elsewhere. While the increasing centralisation of health care services [33] is supported by evidence that the best outcomes are obtained when patients are treated by practitioners and institutions with high caseloads, [34] this centralisation has implications for rural cancer patients' access to diagnostic and treatment services. Typically rural patients need to travel longer distances to access those services, and this may be a disincentive to undertake or complete treatment regimens. This is particularly relevant when the treatment involves a prolonged absence from home resulting in disruptions to family life and financial hardship [35].

#### Indigenous status

While the incidence of breast cancer in Indigenous Australian women is lower than that of non-Indigenous women, it is still the most common cancer in this group [6,36]. Once diagnosed with breast cancer, Indigenous women have significantly poorer survival than their non-Indigenous counterparts [10]. One factor contributing to this disparity is that Indigenous Australians are significantly more likely to live in rural and remote areas than are non-Indigenous Australians [37].

#### Socioeconomic status

In Australia the incidence of breast cancer is about 15% higher for women living in areas of high SES compared to those living in the most disadvantaged areas [6]. Similar patterns have been observed in the USA and Europe with incidence rates about 10-20% higher for women living in the least deprived areas [38-40]. However, women in disadvantaged areas have poorer survival and higher rates of recurrence. While some of the survival differential is explained by stage of disease, significant differences in survival remain after adjustment for tumour size, stage, age at diagnosis and treatment [41,42]. In a recent study examining both area- and individual-level SES, after adjustment, there remained a persistent association for higher mortality for women who lived in communities where a high proportion of residents had limited educational attainment [43].

SES also influences choice of treatment with women from lower SES groups significantly less likely to undergo breast conserving surgery independent of disease stage and less likely to undergo or to complete adjuvant therapy despite this being the recommended treatment [44,45]. Lower SES has also been shown consistently to predict poorer psychological outcomes after cancer [46].

### Area-level versus individual- level factors

Most studies examining area variation in cancer outcomes have used an aggregate ecologic design [47,48]. This has well-documented conceptual and statistical difficulties in interpretation [49]. Survival studies using aggregated ecologic data are unable to quantify whether the variations in survival are likely due to the clustering of individuals or the environmental characteristics of the areas in which the individuals live. Ecologic studies leave open the possibility that variation between clinical and psychosocial outcomes is explained by varying population compositions, and unless these are taken into account, area- and individual-level sources of variation are confounded.

By obtaining detailed information about individual breast cancer patients through clinical records, telephone interviews and self-administered questionnaires, supplemented by area-specific information, this study will incorporate a multilevel approach to investigating inequalities in outcomes, and thus overcome many of the methodological and interpretive problems of ecologic studies.

### Study Aims

1. To systematically describe modes of presentation, time to diagnosis, diagnostic and treatment pathways, utilisation of psychosocial care services, unmet supportive care needs, perceptions of control over treatment decisions, rates of recurrence and survival for women newly diagnosed with breast cancer.

2. To examine differences in these factors according to individual characteristics (including age (< 50 years, 50-69 years and 70+ years), spread of disease, co-morbidity and individual-level socioeconomic status) and area-level characteristics including geographical remoteness, area socioeconomic disadvantage.

3. To model the independent contribution that area-level and individual-level factors make to the variation in recurrence and survival from breast cancer.

## Methods/Design

### Funding and support

This project was awarded funding by Cancer Australia (ID: 1006339). Cancer Council Queensland provided additional funding for the maintenance of the GIS software and for the long-term follow-up of breast cancer cases.

### Ethical clearance

Approval for the study was obtained from the Human Research Ethics Committee of Griffith University, Australia (PSY/C4/09/HREC). Additional clearance to access confidential health information was obtained from the Research Ethics and Governance Unit, Queensland Health.

### Study Design

This study involves the collection and modelling of individual- and area-level data. Individual-level data will be obtained through a longitudinal study of women newly diagnosed with breast cancer. Quantitative and qualitative data will be collected by Computer Assisted Telephone Interview (CATI) and self-administered questionnaires (SAQ) at two time points: 4 to 6 months post diagnosis (Time 1) and 18 months post-diagnosis (Time 2). Clinical and treatment information will be obtained from medical records at 12 months post diagnosis. Information on mammographic screening, surgical and other therapeutic procedures will be obtained from BreastScreen Australia, Medicare Australia and through the Queensland Hospital Admitted Patient Data Collection (QHAPD). Information on recurrence and survival will be obtained using Queensland and interstate Cancer Registries and the National Death Index. Area-level data will be obtained from the Australian Bureau of Statistics.

### Setting

The study will be conducted in Queensland Australia. Queensland is the third largest Australian state by population with an estimated resident population of 4.5 million (2010). Queensland is the most decentralised state in Australia with approximately 40% of its population living outside of its capital city and surrounding area [50].

### Study participants

Eligible participants will be all women aged 20 to 79 years resident in Queensland, Australia with a histologically confirmed diagnosis of invasive breast cancer (ICD0-3 C50) between January 1 2011 and December 31 2012 and notified to the Queensland Cancer Registry (QCR). The QCR is a population-based register with virtually universal coverage of individuals diagnosed with cancer in Queensland. Notification of cancer is a statutory requirement for all pathology services, private and public hospitals and nursing homes in Queensland.

Based on Queensland incidence rates, [9,51] and allowing for increasing trends in counts, [9] it is anticipated that approximately 5, 185 women will be eligible. Patients' name and the names of their treating doctors will be obtained from the QCR using a rapid ascertainment procedure that will minimise the time from diagnosis to ascertainment to approximately four months. Following doctor's consent, all patients will be forwarded a study information sheet, consent form and reply-paid envelope along with a letter from their doctor informing them of the study. Participants will also be asked to provide consent for researchers to access their records within the QCR and to allow matching of their name to other data sources. Non-responding patients will be followed-up by repeat mailing and telephone.

### Data collection

#### Individual-level data

##### Information obtained from telephone and self-administered questionnaires

Participants will receive a structured telephone interview and a self-administered questionnaire (SAQ) at 4 to 6 months (Time 1) and at 18 months (Time 2) post diagnosis. Information collected will include socio-demographics, self-reported diagnostic and treatment information, medical information, attitudes to help seeking, utilisation and availability of psychosocial and supportive care, psychological distress and quality of life.

*Socio-demographics *will include education, marital status, occupation, private health insurance, gross household income (pre and post diagnosis), number of dependent children at home and Indigenous status.

*Medical history *will include family history of breast cancer, birth weight, age at menarche, menopausal status, parity, history of breastfeeding, use of oral contraceptives and hormone replacement therapy (HRT), complementary therapies, current weight, pre-diagnosis weight, pre-existing medical conditions, smoking, and alcohol consumption and levels of physical activity.

*Pathways to diagnosis *will include history of mammography (through public and private facilities), symptoms and date of first recognition of abnormality (for symptom detected), date of mammography (for screen detected), date and outcomes of first appointment with doctor, date and outcomes of any subsequent appointments up to the date of diagnosis, methods of transport and time taken to attend diagnostic tests, satisfaction with the process of diagnosis (including perceived delays, reasons for delays).

*Treatment pathways *will include treatment types and corresponding dates, location of treatment facility, time taken and method of transport to attend treatments, satisfaction with medical care and health system, reasons for treatment choices, perceived degree of control over choices and barriers from the patient's perspective.

*Psychosocial outcomes *will be assessed using a number of previously validated instruments including the *Attitudes to Seeking Help after Cancer (ASHCa) *a 20 item scale that assesses positive and negative attitudes to seeking emotional or psychological support after cancer and behavioural intention to seek support [31,52]. *The Supportive Care Needs Survey Short Form 34 (SCNS-SF34) *a 34-item survey assessing cancer patients' need for help over the last month across 5 domains: psychological, health systems and information, patient care and support, physical and daily living, and sexuality needs. It has well demonstrated reliability and validity in cancer populations [53]. *Perceptions of control over treatment choices *will be assessed using questions developed by Street and Voigt [54] and Jansen et al. [55]*The Brief Symptom Inventory - 18 (BSI-18) *will be used to assess psychological distress through three subscales of anxiety, depression, and somatisation. This scale has been well validated in oncology settings and has also been validated with non-clinical populations in the community [56]. Finally, *Quality of Life *will be assessed using the FACT-B, which has good reliability and validity and has been used in rural patients and among breast cancer survivors in Australia [57,58]. The FACT-B assesses physical, social, emotional and functional well-being.

##### Queensland Cancer Registry Data

Information obtained from the QCR will include date of birth, address at diagnosis, treating physician's name and contact details, date of diagnosis, tumour site, morphology, histological grade, stage, degree of lymph node involvement, oestrogen and progesterone status. Indigenous status will also be collected from QCR records and will be cross-checked against information provided by the participant during the telephone-based interview.

##### Medical record data

At 12 months after diagnosis, information on diagnosis and treatment including diagnostic tests, presenting symptoms, type and date of surgical procedures, post-operative complications and other therapy (radiation, cytotoxic or hormonal therapy), including start and completion dates will be extracted from clinician and hospital records. Information on indicators of illness status and disease progression including stage, tumour site, maximum tumour diameter, lymph node status, presence of distant metastases and other prognostic indicators will also be obtained. Mammographic history (including dates, location and results) will be obtained from the relevant public or private breast screening services.

##### Medicare information

Australia's universal health care system (Medicare) provides access to free treatment at a public hospital and free or subsidised treatment by medical practitioners and specialists. Following additional consent from study participants, data relating to specialist attendances, surgical and therapeutic procedures, and pharmaceutical usage will be extracted from the Medicare database from 1^st ^January 2011 for the next five years.

##### Long-term follow-up of participants

Using the QCR, other Australian state and territory cancer registries, and Australia's National Death Index, participants will be followed for a total of five years from their date of diagnosis for breast cancer recurrence, diagnosis of other primary cancers, and death (classified as breast cancer, other cancer or non-cancer death).

#### Area-level information

Statistical local areas (SLA) will be the primary focus for area-level analysis. SLAs comprise a group of census collection districts (CDs), the smallest geographical area used by the Australian Bureau of Statistics. In 2006 there were 478 SLAs in Queensland with a median population of 5, 810 individuals. SLAs are often based on the incorporated bodies of local governments who are responsible for service provision and infrastructure at the local and regional level.

##### Remoteness

Remoteness of residence when diagnosed with breast cancer will be categorised using the ARIA+ classification, which is a measure of accessibility and remoteness based on geographical location [59]. This classification includes Major City, Inner Regional, Outer Regional, Remote and Very Remote.

##### Access to cancer treatment centres and medical care

As the ARIA+ classification does not account for access to specialised treatment facilities, specific categories based on distance to and from each facility will be developed. Distance to treatment facilities will be calculated from the participant's place of residence. Address details for breast cancer patients, treatment facilities and diagnostic services will be geocoded using the Manifold^® ^GIS System, a commercial package designed to clean and convert address information into latitude and longitude coordinates. Road travel distances and times between these locations will be calculated using Manifold^® ^GIS system, combined with street network analysis and custom GIS applications.

##### Measures of socioeconomic status

Area-level socioeconomic disadvantage will be measured using the Index of Relative Socioeconomic Disadvantage (IRSD) calculated by the Australian Bureau of Statistics (ABS) [60]. The IRSD provides a general measure of disadvantage, and considers factors such as low income, low educational attainment, high unemployment, and jobs in unskilled occupations. Area-level information about education levels, household income, types of occupation, median mortgage and rental payments and other socioeconomic indicators (corresponding to the relevant information collected at the individual level) by age group and sex for each SLA will be obtained from the census data files released by the ABS for 2006 and 2011.

##### Life expectancy

Average life expectancy will be calculated from the unit record mortality file for Queensland from the ABS that contains year-specific details of all-cause mortality by SLA.

#### Primary outcome measures

Diagnostic pathways will be assessed through the collection of information describing events and the time period from participants' first noticing symptoms or first abnormality on routine screening exam to first presentation to the medical practitioner. The diagnostic interval will be calculated as the time between initial presentation and the definitive diagnosis.

Treatment pathways will include a description of events and the time taken from definitive diagnosis to the commencement of primary treatment and then the time taken from primary treatment to subsequent treatment(s).

Time to recurrence will be calculated from the date of initial diagnosis to the date of first recurrence. Survival will be calculated from the date of initial diagnosis to the end of the follow-up period (five years) or death.

#### Sample size and power calculations

Based on the number of women diagnosed with breast cancer in Queensland from 2005-2007, [51] and recent trends in incidence counts, approximately 5, 185 women aged 20 to 79 years at diagnosis will be eligible for the study. Survival with breast cancer in Queensland is 95.5% one year after diagnosis and 91.3% at two years. Assuming that, doctor's permission is obtained to contact 85% of patients, and 75% of these patients agree to participate with an additional 5% loss-to-follow up in the second year of the study, the actual sample sizes would be 3, 305 at time 1 and 2, 985 at time 2.

The 5-year cause-specific survival for women in Queensland diagnosed with invasive breast cancer when living within 2 hours road travelling time from a radiation facility is 0.897, compared to 0.851 for women living further away (unpublished data, QCR). Applying these estimates to the anticipated sample size (n = 2982; n = 2289 < 2 hrs; n = 692 ≥2 hrs) and using a two-sided log rank test, we would expect 80% power at a 5% significance level to detect an absolute survival difference of 4%, or an approximate hazard ratio of 1.4. This equates to 356 breast cancer deaths expected among the study cohort within 5 years. Power calculations for the variation between and within area units (using *Optimum Design *software) are based solely on the approximate number of clusters (i.e. SLA) and records per cluster. We expect the sample at time 2 to cover 418 area units (SLAs), with between 1 and 69 (mean 7.4, median = 5) respondents in each unit. Assuming 400 clusters with an average of 7 records per cluster and a baseline proportion of 50% gives 85% power at 0.05% significance to detect a difference in proportions between two groups of 6% at time 2.

#### Statistical analysis

Statistical analyses will be conducted using a combination of standard empirical procedures and multilevel modelling (MLwiN 2.23). Time to diagnosis and treatment will be analysed using standard methods such as geometric mean (to account for anticipated skewed distribution of time to diagnosis and treatment) or the median.

Multilevel modelling (Cox proportional hazards) includes individual-level factors (level 1) and the indicators of geographic remoteness and area socioeconomic disadvantage (level 2). This approach extends traditional (single-level) proportional hazards modelling by incorporating a random intercept that reflects the average survival outcome (time or probability) for each area. Corresponding models will be used for count (based on the Poisson or Negative binomial distribution) and categorical (based on the binomial distribution) data.

The multi-level modelling strategy consists of three stages. Firstly, building on the null (intercept only) model, this includes individual-level factors (such as patient characteristics, disease stage, co-morbidity, and access to health care and treatment services) as fixed effects. This will tell us how much of the area-variation in the breast cancer outcomes is due to these compositional factors; and also assess the contribution of each individual-level factor, including age to these outcomes. The second model extends Model 1 by including the area-level variables such as geographic remoteness and area socioeconomic disadvantage as fixed effects. This will quantify how much of the area-variation in the diagnostic pathway, treatment choices, unmet needs, and psychological adjustment and recurrence and survival is due to the area-level factors, independently of the individual-level factors. In the third model we specify cross-level interactions between the individual-level factors and geographic remoteness and area disadvantage. This analysis will tell us if the relationship between breast cancer outcomes and each of the individual-level factors differs as a function of geographic remoteness or area disadvantage.

## Discussion

Breast cancer is the most common cancer in women in Australia. As the population increases and ages, the number of women diagnosed with breast cancer will increase, increasing the already significant burden on health care services. Inequalities in clinical and psychosocial outcomes, including poorer survival continue for women from rural and regional areas and for women from areas of disadvantage. The study will use an innovative approach to investigate reasons for these inequalities. Applying multi-level modelling techniques to examine area-level factors simultaneously with detailed individual-level factors will enable us to build a more complete picture of what are the important contributors to these known inequalities and how these factors relate to each other.

This study will enable us to determine whether geographical areas themselves have an impact on outcomes from breast cancer independently of the characteristics of the women who live in these areas. It will provide health care providers and policy makers with targeted information to improve the future management of women with breast cancer, and inform the development of strategies to improve psychosocial care for women with breast cancer.

## Competing interests

The authors declare that they have no competing interests

## Authors' contributions

PHY led the writing for this manuscript and wrote the introduction, measures, and discussion. PDB wrote sections relating to the multilevel modelling and sample size components. All authors contributed to the design and data collection protocols, and wrote sections of the grant application that was used in the paper. All authors read and approved the manuscript.

## Pre-publication history

The pre-publication history for this paper can be accessed here:

http://www.biomedcentral.com/1471-2407/11/415/prepub
